# Supplementary material for: Staying With the Trouble in Nursing 12.5 Hours at a Time
Source: Nurs Philos. 2025 Dec 17;27(1):e70060. doi: 10.1111/nup.70060 (PMC12711605; doi:10.1111/nup.70060)
Supplement: Supplementary file 1 — SPIN event of nursing R1 Tracked2. [file NUP-27-e70060-s001.docx]

# Staying with the Trouble in Nursing

## Abstract

This paper proposes a reconceptualisation of clinical care as a site from which philosophy is produced, rather than a domain to which it is applied. Centering on a speculative vignette drawn from emergency nursing, I introduce the concept of the *event of nursing* to name those moments where relational, material, and institutional forces exceed protocol and call for situated, embodied ethical judgement. Through this lens, nursing practice is not the application of philosophical ethics, but an already-theorising practice; one that enacts, improvises, and sustains a posthuman ethics in real time. Drawing on Spinoza, Barad, Haraway, Braidotti, and Deleuze and Guattari, I argue that nursing’s entangled thinking-feeling-doing constitutes a form of knowledge irreducible to either abstraction or procedure. I frame response-ability as the immanent composition of powers (conatus), read diffractively, tracing how care is produced through the navigation of competing temporalities, machinic infrastructures, and affective pressures. With the risks of epistemic violence of algorithmic governance and linear reasoning, I offer three speculative propositions: ‘assemblage notes,’ ‘situated adequacy metrics,’ and ‘posthuman night rounds.’ These gestures aim not to reform systems but to make legible the improvisational labour that sustains care amid constraint. The event of nursing, I suggest, offers philosophy a model of ethics grounded in affective intelligence, situated multiplicity, and infrastructural friction, a way of staying with the trouble, twelve hours at a time.

**Keywords**

posthuman nursing

new materialism

nursing philosophy

nursing ethics

diffractive reading

response-ability

event of nursing

## Response-ability at 4 AM: A Composition Forms

At 4:18 on a Tuesday in February, Mr Jones presents to A&E with an infected diabetic foot ulcer^^[[1]](#footnote-1)^^. The triage system assigns him green: non-urgent. This categorisation, following established protocols, creates a four-hour wait minimum. Yet Maria, the nurse covering additional bays due to staffing shortages, recognises a familiar pattern. Cues across gait, the way he protects his rucksack, the specific quality of his limp - these details reconfigure the initial triage category. In this recognition, something begins to happen that exceeds the routine application of clinical knowledge. An event is forming.

The context of Mr Jones’ presentation compounds its complexity. Police cleared his sleeping place six hours ago; he has been walking since. Each step looks painful and is no doubt adding to an already compromised situation. The wound itself presents clear medical indicators: 4 cm x 3 cm ulceration with purulent discharge, undermined edges, and cellulitis tracking proximally. Standard protocols are unambiguous. National guidelines mandate aggressive intervention: wound culture, IV access, immediate antibiotics, admission for intensive treatment. Yet these evidence-based recommendations assume certain conditions: stable housing, access to medication, the ability to elevate the affected limb, and regular follow-up appointments.

Mr Jones's material reality creates different parameters. "Can't stay," he states before Maria asks. His belongings remain hidden but vulnerable. This calculation—medical treatment versus losing essential possessions—shapes every healthcare encounter for those people sleeping rough (Hewett et al., 2012). The conflict isn't simply between compliance and non-compliance, but between competing survival needs that healthcare systems rarely acknowledge (Marmot et al. 2020; Browne et al. 2015). Here, in this gap between protocol and possibility, the event of nursing is produced. Rather than using philosophy to explain what nursing does, this paper insists that nursing already enacts the core commitments of new materialist and critical posthuman ethics. Theory arrives late to a scene that nurses have been improvising daily. This reversal is not a rejection of theory, but a repositioning: placing practice as the ground from which concepts are in production.

Multiple temporalities converge in this moment. The four-hour emergency target exerts institutional pressure toward rapid discharge. Bacterial reproduction follows its own timeline, with infection advancing steadily through tissue. Mr Jones's anxiety about his abandoned belongings creates another urgency. The encounter composes with competing temporal demands while the wound continues to deteriorate. Standard intervention promises clinical improvement but almost guarantees self-discharge. No intervention risks progression to sepsis within days. The event of nursing happens precisely here: in the navigation of irreducible multiplicities that cannot be resolved through protocol alone. Such work is neither rare nor heroic; it is ordinary night‑shift craft.

I align my arguments with a growing strand of nursing philosophy that foregrounds care as relational, situated, and ethically complex (Tronto, 1993; Nelson & Gordon, 2006). This also resonates with recent posthuman and feminist interventions into healthcare, particularly those that resist the abstraction of practice into disembodied protocols (Sellman, 2011; Bender & Elias, 2021). While the theoretical framework draws on Spinoza, Barad, Braidotti, and Deleuze and Guattari, it builds on a lineage of nursing thinkers who have long argued for the epistemological legitimacy of practical wisdom, clinical intuition, and embodied judgement.

In this paper, I contribute to nursing philosophy in three key ways. First, I propose a reconceptualisation of clinical care not as an application of theory but as a generative site for philosophical thought. Second, I introduce the concept of the “event of nursing” to name the entangled, situated, and often fugitive production of care under pressure. Third, I offer three speculative propositions that imagine infrastructural shifts aligned with posthuman ethics. In doing so, the paper bridges philosophical abstraction and clinical particularity, inviting new ways of perceiving and supporting nursing practice amid algorithmic constraint.

## The Philosophical Stakes

This moment exemplifies what Haraway (2016) calls "staying with the trouble" - not as a philosophical abstraction, but as a practical necessity. Maria faces a situation where established protocols and material realities create interference patterns rather than clear pathways. The electronic health record awaits input, its dropdown menus offering standardised options that fail to capture the assemblage forming in cubicle seven. What happens in this space between institutional categories and lived complexity is the event of nursing itself: always singular, never routine, demanding response-ability rather than merely applying knowledge.

Traditional Western epistemology would demand Maria choose: follow evidence-based protocols or trust situated intuition. This binary framework assumes that knowledge exists in discrete categories of scientific or experiential, objective or subjective, universal or particular. But Maria's response-ability operates through what Deleuze and Guattari (1987) term "and-and" logic. She holds multiple knowledge systems in productive tension without resolving them into a singular or fixed hierarchy. The protocols remain valid; years of evidence support their efficacy. Her embodied knowledge of homelessness and healthcare remains equally valid; fifteen years of night shifts have taught her patterns no randomised controlled trial can record. Neither cancels the other; together they create diffractive patterns that reveal new possibilities for action.

This is not indecision or relativism. Maria's hesitation - those crucial seconds before acting - represents what Barad (2007) calls an "agential cut," a moment where response-ability is produced through the entanglement of multiple agencies. Adequacy here is internal coherence of causes (Spinoza, 1996, IIP40S2). The wound speaks its biological truth through inflammation markers and bacterial load. The institution speaks through waiting time targets and admission criteria. Mr Jones's life speaks through the careful positioning of his rucksack, his calculation of risks. Maria doesn't synthesise these voices into false harmony. She navigates their interference patterns, reading where they amplify or cancel each other, finding paths that honour their irreducible multiplicity.

The stakes extend beyond this singular encounter. As healthcare increasingly turns toward algorithmic decision-making and standardised pathways, the capacity for such navigation becomes endangered (Smith et al 2023). Algorithms excel at applying protocols consistently but cannot read interference patterns between knowledge systems. They process either-or but cannot think and-and. What Maria embodies - this cultivated response-ability to hold multiple realities without resolution - represents a form of clinical wisdom that resists codification yet remains essential to ethical care.

### The Intervention

This paper proposes reading feminist new materialism from a different perspective: not using theory to illuminate practice, but recognising how the event of nursing and care already enacts what philosophers theorise. When Barad (2007) describes intra-action, where agencies are produced through encounter rather than preceding it, nurses recognise their daily experience of becoming-nurse-with-patient-with-institution. When Haraway (2016) urges staying with trouble, nurses reply: "We do, twelve hours at a time." When Braidotti (2013, 2019) maps posthuman subjectivity as embedded, embodied, and transversal, she describes what every nurse navigates when thinking with monitors while reading bodies, following protocols, and creating workarounds. The backwards reading reveals something crucial: it reverses the usual academic order of operations.

Response-ability is produced because standardised responses fail. Assemblage thinking develops because patients span and speciate across available categories. In Braidotti's terms, they manifest as nomadic subjects,transversal multiplicities that evade fixed codification, simultaneously becoming across human and more-than-human dimensions (Braidotti, 2013, p. 56; Braidotti, 2019, p. 54). As Barad (2007) describes, diffractive practices involve reading patterns of difference through their entanglement rather than isolating variables. Diffractive practices evolve because linear pathways reach dead ends. In clinical practice, this might look like a treatment protocol that assumes housing security, or follow-up appointments that presume access to transport, logics that break down when confronted with the complex realities of lives like Mr Jones’s. Diffractive practice, here, involves recognising how protocols, material conditions, professional judgement, and patient experience all interfere with one another to produce situated possibilities for care. Rather than seeking alignment or clarity, diffraction reads these overlaps and tensions as productive, generating pathways through, not around, complexity. Maria doesn't choose posthuman subjectivity; she becomes it through repeated encounters with irreducible complexity. This suggests that care work, particularly nursing, offers philosophy a functioning model of new materialist ethics in practice.

## Spinoza in Scrubs, Monism as Assemblage Practice

This section turns towards the seventeenth-century philosopher Baruch Spinoza to deepen the analysis of what Maria’s practice reveals. While the previous discussion drew on feminist new materialism and critical posthumanism, particularly Barad’s diffraction, Haraway’s "staying with the trouble," and Braidotti’s "nomadic ethics", Spinoza offers a foundational metaphysics that helps make sense of Maria’s refusal to divide thinking from doing, knowing from touching. His monism provides the conceptual ground for understanding why nursing knowledge is not simply applied theory, but a mode of entangled reasoning that is embodied through encounters, challenging the Cartesian dualisms that still organise medical knowledge and institutional decision-making. Long before the philosopher explicitly enters the frame, his refusal to divide mind and body, thought and extension, is already present in the nurse's way of knowing.

The hospital's decision-making apparatus often defaults to binary logics, not because clinical reasoning is inherently dialectical, but because institutional structures require categorisation and resolution. This isn’t a philosophical commitment to synthesis, but a pragmatic demand for computability and auditability. Yet what Maria encounters resists such closure, and requires a different logic: one that holds multiple truths simultaneously without forcing resolution (Whitehead, 2014; Cohen, 1995). This division manifests institutionally: medical knowledge is primarily associated with diagnosis and abstract reasoning, while nursing practice is often framed as the physical implementation of medical orders. The hierarchy is both epistemological and professional. Medical knowledge, coded as cerebral and abstract, claims majoritarian status through its alignment with scientific rationality. Nursing knowledge, understood as manual and experiential, occupies a minoritarian position - necessary but subordinate, practical but not theoretical (Nelson & Gordon, 2006). Protocols crystallise this split, presenting as pure reason extracted from the messiness of embodied encounter, promising to transcend the variability of individual bodies through standardised pathways.

Yet observe Maria as she examines Mr Jones's infected foot at 4:30 AM. Her hands read the wound while her eyes scan lab results on the computer screen. She thinks-feels the temperature differential between infected and healthy tissue, a sensation that carries meaning accumulated through thousands of similar touches. The electronic health record displays white cell count: 14.2 × 10⁹/L - elevated but not dramatically so. Her fingers detect the subtle bogginess that suggests deeper tissue involvement than the numbers indicate. This isn't alternation between thinking and feeling but their simultaneous operation, creating what Barad (2007) calls "interference patterns" - places where different ways of knowing overlap and interact without cancelling each other out.

This simultaneous thinking-feeling challenges the very foundations of Cartesian dualism. In the *Ethics*, Spinoza insists that "the human mind is the idea of the human body" (Spinoza, 1996, IIP13), not as a correlation but as two attributes of the same substance. Thought and extension are not separate realms requiring coordination, but different modes through which substance expresses itself. For Spinoza, the mind thinking and the body acting are "one and the same thing, which, now under the attribute of thought, now under the attribute of extension, is conceived" (Spinoza, 1996, IIIP2S). In Maria's clinical encounter, this monism lives. Her knowledge of infection doesn't divide between abstract understanding and embodied recognition - it is produced through their entanglement. When she palpates the wound edges, she simultaneously thinks-feels: the textbook parameters of cellulitis (erythema, warmth, swelling, pain) diffract through her fingertips' learned sensitivity to tissue resistance. The interference pattern created reveals something neither pure reason nor pure sensation could access alone. This is what Deleuze, reading Spinoza, identifies as "a single substance for all attributes" (Deleuze, 1988, p. 58) - not the collapse of distinctions but their productive entanglement.

Braidotti recognises in Spinoza "a philosophy of radical immanence" (Braidotti, 2013, p. 56) that refuses transcendent divisions between mind and matter, sacred and profane, human and nature. Maria embodies this immanence, not through philosophical commitment but through practical necessity. It is here, in this entangled moment of simultaneous thinking-feeling, that the event of nursing reappears, not as a rupture, but as a manifestation of what Spinoza would call adequate understanding: knowledge emerging from within the complex material conditions of care. The wound presents as an assemblage: bacterial activity, inflammatory response, tissue breakdown, glycaemic control, vascular compromise, and social circumstance. To read it adequately requires thinking that moves across attributes without privileging one over others. Her response-ability is produced precisely from this refusal to separate what the Cartesian hospital would divide: the clinical data from the lived experience, the protocol from the particular, the mind that diagnoses from the body that touches.

### Adequate Ideas as Interference Patterns

Spinoza defines adequate ideas as those that comprehend their object through its full causal modes rather than through partial or confused perception (Spinoza, 1996, IIP40S2). In the contemporary hospital, evidence-based protocols aim at adequacy by tracing consistent causal patterns across populations. However, for Spinoza, adequacy must arise through internal and situational coherence, not external generalisation, suggesting that what counts as 'adequate' in institutional terms may fall short in singular practice (Rhodes et al., 2017). When implemented systematically, these protocols save lives - their adequacy is not in question at the population level.

But at 4:35 AM, as Maria considers Mr Jones's presentation, a different problem of adequacy becomes perceptible. The protocol assumes certain conditions: that the patient can remain in the hospital, that IV access can be maintained, and that follow-up is possible. It achieves adequacy through abstraction from particulars. Yet Mr Jones's reality includes hidden belongings growing more vulnerable each minute, previous experiences of having his possessions stolen during admission, and the knowledge that morning discharge means nowhere to go. These aren't external additions to a clinical picture but part of what Spinoza would call the "infinite attributes" through which substance expresses itself (Spinoza, 1996, IP11).

Maria's response-ability gathers multiple threads into an adequate understanding: the progression of cellulitis in diabetic tissue (medical knowledge), the specific quality of Mr Jones's agitation (embodied perception), the bed manager's hovering presence (institutional pressure), the police sweep patterns in the area (social context), the types of antibiotics that don't require refrigeration (material constraints). Each thread carries its own validity, its own claim to truth. Adequate knowledge here cannot mean choosing the most important thread but comprehending their entanglement. This resonates with Haraway's insistence that "situated knowledges require that the object of knowledge be pictured as an actor and agent, not a screen or a ground or a resource" (Haraway, 1988, p. 592). The wound speaks its biological truth through inflammatory markers. The institution speaks through audit trails and targets. Mr Jones's life circumstances speak through his protective grip on his rucksack. None of these knowledges are complete; all are partial and located. Yet Maria cannot wait for a god's-eye view that would resolve their differences. Adequacy, in this moment, means something more modest and more radical: sufficient comprehension for response-able action.

The entanglement becomes an epistemological method. Rather than hierarchising knowledge systems, privileging either evidence-based protocols or clinical intuition, Maria allows them to create interference patterns. Where the protocol says "admit" and experience says "he'll leave," she doesn't choose but reads the pattern created by their interaction. This is what Barad (2007) calls "diffractive reading", which attends to patterns of difference when knowledge systems encounter each other.

Braidotti's critical posthumanism emerges here not as a distant philosophical lens but as a practical logic that shapes how Maria navigates the complexities of care. As Braidotti (2019, p. 54) writes, "The posthuman critical thinker has to be worthy of the present," meaning capable of inhabiting and responding to complexity without reducing it. Maria does precisely this: her adequacy arises through a kind of nomadic movement, traversing between majoritarian protocols and minoritarian knowledges, letting each inform the other. This practice of holding tensions without resolution, and of reading interference rather than seeking synthesis, is not just posthumanism in action; it is a clear manifestation of the event of nursing itself.

### Conatus as Assemblage Forces

For Spinoza, every entity possesses conatus, the striving to persevere in being and enhance its capacity of acting (Spinoza, 1996, IIIP6). This isn't mere survival, but an active force toward flourishing, what Deleuze calls "a degree of power" that expresses itself (Deleuze, 1988, p. 97). In the assemblage forming around Mr Jones's infected foot, multiple conatus vectors converge, each legitimate in its striving, creating a complex field of forces that cannot be resolved through hierarchical ordering.

Mr Jones's body expresses its conatus through inflammatory cascades and tissue repair mechanisms, striving to contain infection despite compromised circulation. The bacteria assert their own drive to flourish, multiplying in the glucose-rich environment of diabetic tissue. These biological forces operate independently of human intention, what Bennett (2010) calls "thing-power" - the active, productive capacity of matter itself. Maria's professional conatus drives toward healing, toward enhancing life possibilities, shaped by training and experience into particular patterns of response. The institution's conatus is expressed through efficiency protocols, bed management systems, audit mechanisms, striving to maintain flow, reduce bottlenecks, and meet targets.

These forces don't oppose each other in simple antagonism; indeed, opposition is not a necessary condition for taking a position. Mr Jones's need to protect his belongings expresses a conatus toward maintaining the material conditions for survival on the streets. This doesn't contradict his body's need for medical treatment but creates what Deleuze and Guattari (1987) term "assemblage" - heterogeneous components held together through relations of exteriority. The antibiotic protocol's drive toward standardised care doesn't negate Maria's embodied wisdom but enters into complex relations with it. Each force maintains its trajectory while being affected by the others.

Response-ability becomes the capacity to navigate these converging forces without cancelling their legitimacy. Maria doesn't choose which conatus matters most - a hierarchical solution that would privilege one force over others. Instead, she engages in what Braidotti calls "nomadic ethics" - "a matter of forces and levels of intensity, a process ontology that supports multiple modes of relation" (Braidotti, 2013, p. 92). She reads how the forces interfere with each other: how Mr Jones's anxiety about his belongings intensifies when admission is mentioned, how this anxiety affects his autonomic responses, potentially complicating clinical assessment. She notes how institutional pressure toward rapid discharge resonates with his desire to leave, creating a convergence that could enable a workable solution.

Clinical wisdom here means developing sensitivity to these interference patterns. Maria has learned, through repeated encounters, how different forces create different possibilities when they interact. She recognises that working with, rather than against, these patterns often produces better outcomes - not optimal by any single measure, but sustainable across multiple dimensions. When she creates a treatment plan that acknowledges Mr Jones's need to return to his belongings while addressing the infection, she's not compromising medical standards but finding a path through converging forces.

This is assemblage work as ethical practice: holding space for multiple legitimate strivings, reading their patterns of interference and amplification, navigating toward solutions that enhance rather than diminish collective possibilities. It embodies what Braidotti (2019) envisions as posthuman subjectivity; away from the axiom of the liberal individual choosing between options rather the nomadic subject constituted through and navigating between multiple relations, always in the middle of forces that exceed and constitute it simultaneously.

## Beyond Dialectics: And-And Assemblages

Building on Spinoza’s monism and the concept of adequate ideas, this section explores how Maria’s clinical reasoning extends further through the Deleuzian logic of proliferation. Where Spinoza offered the grounding for thinking-feeling as a simultaneous, entangled mode of knowing, Deleuze and Guattari provide a means for understanding how the event of nursing unfolds through transversal connections and irreducible multiplicity. If the event, as we have seen, resists binary resolution, then Deleuze's "and-and" logic shows how that resistance becomes method, how Maria acts not by choosing between competing demands, but by composing a care practice from their friction.

The clinical event extends. Maria has recognised Mr Jones's wound as more than infection: it exists within an assemblage of homelessness, institutional demands, and biological urgency. Yet the hospital's decision-making apparatus operates through dialectical resolution. Either follow the sepsis protocol (thesis) or accommodate social circumstances (antithesis), seeking some synthetic middle ground that satisfies neither medical necessity nor material reality. This dialectical framework, deeply embedded in clinical reasoning, promises resolution through hierarchical integration. But what Maria faces at 4:40 AM resists such neat synthesis.

Healthcare professionals understand this resistance intimately. Both doctors and nurses recognise that clinical reality exceeds binary frameworks through every shift they work. Yet the institutional apparatus creates hierarchies of knowing that valorise certain forms of knowledge while marginalising others. Cognitive, abstract knowledge (diagnosis, pharmacology, pathophysiology) claims premium status through its alignment with scientific modernity. The embodied, social, and situated knowledge that enables care gets acknowledged as necessary but somehow lesser, relegated to the "art" of medicine rather than its "science." This isn't ignorance but rather what Santos (2014) calls "epistemicide": the systematic devaluation of ways of knowing that don't conform to dominant paradigms.

Deleuze and Guattari offer a different logic through their reading of Spinoza. Where dialectics seeks synthesis, they propose a proliferative logic of "and... and... and..." (Deleuze & Guattari, 1987, p. 25). This isn't movement toward resolution but what Deleuze, in his solo work on Spinoza, describes as terms becoming "a medium for a movement that relates it to the other term" (Deleuze, 1988, p. 122). The protocol remains fully present with its evidence base and life-saving potential. Mr Jones's need to protect his belongings maintains its existential urgency. The institution's four-hour target continues to exert temporal pressure. None cancels or subsumes the others; instead, they create what Barad (2007) calls "diffraction patterns", interference that reveals new possibilities without erasing differences.

Consider how Maria navigates this multiplicity. She doesn't choose between competing goods or seek a compromise. Instead, she allows different logics to interfere productively. The sepsis protocol says: culture wound, establish IV access, administer broad-spectrum antibiotics, admit for observation. Fifteen years of night shifts with homeless patients say: he'll self-discharge within hours, losing both treatment and trust. The institution demands: meet targets, document decisions, manage flow. Each logic remains valid within its own terms, creating not contradiction but what Deleuze and Guattari term "assemblage": heterogeneous components maintaining their differences while producing new capacities through connection.

To deepen this logic of multiplicity and non-resolution, Deleuze and Guattari introduce the figure of the rhizome. Unlike the tree, which branches from a central trunk in hierarchical sequences, the rhizome is in non-linear relation: "it has neither beginning nor end, but always a middle (milieu) from which it grows and which it overspills" (1987, p. 21). This image helps us understand how clinical reasoning might grow through situated connections rather than top-down synthesis. Maria’s way of thinking doesn’t start from fixed categories, it happens in the middle of things.

Her clinical reasoning operates rhizomatically, making connections between wound assessment and police sweep schedules, between antibiotic pharmacokinetics and the materiality of street life, between institutional metrics and embodied suffering. These connections don't follow predetermined pathways but are produced with the encounter itself, creating what Haraway (2016) calls "unexpected collaborations and combinations." In this, we see the event of nursing itself, not as a fixed practice or resolution, but as an ongoing recomposition of possibilities. The and-and logic doesn’t simplify complexity; it sustains it, allowing modes of care to be created from within constraint.

### Assemblage Under Pressure

The rhizomatic logic introduced above becomes even more critical as the clinical assemblage thickens. Before we return to Braidotti’s concept of nomadic practice, it is worth tracing how these shifting relations and recompositions of care call for a subjectivity that is not fixed but continuously becoming. Nomadism, in Braidotti’s terms, is not about displacement but about ethical and political recomposition in response to situated complexity (Braidotti, 1994/2011). At 4:45 AM, a ripple moves through the unfolding assemblage: the bed manager circles the department, the security guard notes the gathering of rough sleepers in the waiting room, the junior doctor seeks guidance, and the electronic prescribing system with its mandatory fields. Each brings its own temporality and demand. These aren’t background details; they’re integral components of the assemblage Maria must now navigate.

The clinical guidelines for diabetic foot infections remain applicable, grounded in systematic evidence and standardised care pathways (National Institute for Health and Care Excellence, 2015). But when these protocols enter the assemblage, they no longer function as standalone truths. They become one force among many, jostling with Maria’s embodied knowledge, the patient's lived reality, and institutional infrastructures. When the protocol demands "urgent surgical referral," but the patient is already halfway out the door, it’s not a failure of logic, but rather an encounter with multiplicity.

Maria doesn't merely hold these contradictions in suspension. She crafts a temporary, functional consistency out of them,a consistency Deleuze and Guattari describe not as order but as provisional coherence. Without this adaptive composition, the incoherence of institutional demands might fracture subjectivity itself. This, perhaps, is Deleuze and Guattari's provocation in *Capitalism and Schizophrenia* (Deleuze & Guattari, 1987): under the pressures of late modern institutions, the subject must constantly recompose itself or risk being torn apart. Maria’s response is neither compliance nor resistance, but what Braidotti (2013) might call transversal ethics in action: connecting across logics, rather than resolving them. Clinical wisdom here means reading the rhythms of pressure: how Mr Jones’s anxiety tightens when admission is mentioned, how that stress alters his clinical presentation, how the pressure to discharge aligns with his own sense of urgency.

Maria’s work is not merely technical; it is affective, temporal, and situated. This is where becoming-nurse begins, not as a title but as a process. Deleuze and Guattari’s “becoming” is never about imitation or completion; it is about transformation through relation. Maria doesn’t simply apply what she already knows. She becomes a nurse again in this particular encounter, reconstituted through this unique configuration of forces. Her documentation habits shift to create administrative space for return visits. Her choice of antibiotics adjusts for non-refrigerated storage. Her assessment unfolds with knowledge that cannot be found in textbooks: the bodily memory of past discharges gone wrong, the social knowledge of who watches whose belongings in the waiting room. Each decision is a minor gesture (Manning, 2016),a subtle adjustment that opens the possibility for survival.

This becoming is not heroic. It is exhausting. Yet Braidotti’s nomadic practice is not only reactive but generative. It involves crafting life-affirming alternatives within constraint, experiments in how to live and act otherwise. Maria’s work, then, is not merely the art of coping; it is the practice of inventing forms of care that endure despite systemic neglect. Her minor gestures are also world-making. They do not merely sustain the present, they open space for a different ethics of nursing, one grounded in situated adaptability, political clarity, and affective intelligence. This is not just nomadic ethics as a means of survival, but also as a speculative resistance. It is not a solution to structural violence, but a form of ongoing resistance within it. As Braidotti writes, the posthuman subject is one who learns to sustain variation: not fixed identity but nomadic movement across demands (Braidotti, 2019). Maria’s subjectivity is not hers alone, it is co-composed with protocols, machines, histories, and lives. Power here is composed immanently; Maria is one mode among many.

What we perceive is not a nurse who solves the unsolvable, but one who navigates it with care. This is what the event of nursing looks like under pressure: not a heroic act or a final answer, but the contingent holding together of competing goods, intensities, and timelines, staying with the trouble twelve hours at a time. In this way, the event becomes a situated ethics: a moment of recomposition that resists both collapse and closure, even if only until morning.

##

## Care Under Constraint: Nomadic Ethics and Algorithmic Friction

The bed manager checks capacity; the security guard continues their rounds; the junior doctor requests guidance. Meanwhile, the electronic prescribing system flashes reminders: antimicrobial protocols incomplete, documentation pending. Each of these entities exerts a different pressure, a different rhythm. These are not background noise-they are constitutive elements of the event. They shape the medium through which Maria must compose care.

Maria doesn’t simply apply knowledge; she becomes nurse again through this encounter. Her gestures are not automatic-they are reconstituted through this specific configuration of urgency, fatigue, institutional logic, and embodied memory. Her documentation shifts to allow space for return visits. Her choice of antibiotics accommodates the reality of street life-heat-stable, once-daily dosing. Her assessment unfolds through a sensitivity trained over years of night shifts and lost patients. These are minor gestures in Manning’s (2016) sense-small acts that open space for ethical possibility. Maria’s care is not heroic, but it is inventive, relational, and exhausting. Yet this form of care becomes increasingly endangered. The EPMA system had defaulted to IV antibiotics. Deviations from protocol trigger alerts. Maria negotiated a clinically appropriate oral alternative with the junior doctor. Supplies are assembled and handed over, along with dressings, glucose tablets, and a spare pair of socks. These decisions sit outside the prescribed flow. Within the system, her actions register as deviant.

Clinical decision support systems (CDSS), embedded in prescribing software, model care through arborescent logic: rule-bound, linear, and hierarchical (Deleuze & Guattari, 1987; Topol 2019; Alsaidan 2022; Lazzarino 2024). IV before PO. Observations before discharge. No prescription without indication. These pathways encode an epistemology that renders situated judgement as risk, and deviation as error. Power flows through these defaults-not by dictating action, but by rendering only some actions legible. Maria’s proceeds with her cares as one causal strand with others. She moves with and through the protocol, drawing on an embodied archive of prior patients, frayed rotas, and silent institutional histories. Her conatus is situated, not transcendent-an effort to continue composing care within constraint. This may be an act of situated ethics, but it is also pragmatic. If Mr Jones returns in two days, septic or worse, she or her colleagues will carry that burden. The speculative wager she makes is ethical, but also strategic, even self-preserving. As Barad (2007) might argue, her act is a diffractive practice: an entangled becoming shaped by material, institutional, and affective relations. A reading that follows the interference patterns-not comparing systems, but tracing how they materialise care.

This is nomadic ethics in action. As Braidotti (2019) writes, the posthuman subject is one who learns to sustain variation-whose subjectivity is recomposed through multiplicity. Maria’s decisions are not drawn from fixed identity or linear reasoning. They are created from an affective and machinic mesh: dropdown menus, fluorescent lights, bodily memory, institutional silence. She doesn’t resist the assemblage; she lets it recompose her. Deviance, in this frame, is not failure. It is a signal. A gesture. A speculative wager. Maria’s wager is that Mr Jones might return, might take the antibiotic, might heal just enough. This is not compliance-it is care’s improvisational tenacity. It is the excess that sustains practice when the dropdown menu ends. Had an assemblage note existed, her decision might still appear deviant in the audit log-but that deviance would be legible as ethical. As conatus. As situated adequacy. The socks, the once-daily antibiotic, the printed plan are not workarounds. They are speculative gestures that made the protocol work. They are the event of nursing.

##

## Speculative Propositions for Posthuman Nursing

If Section 3 named the fragility of Maria’s and‑and practice under algorithmic pressure, this final analytic section moves from diagnosis to affirmative speculation. What conceptual inventions might help us better understand the event of nursing, sustain nomadic ethics, and keep diffraction visible inside digital health infrastructures? Drawing on Braidotti’s call for “life‑affirming alternatives within constraint” (2019) and Haraway’s insistence on making kin with technology, I offer three tentative propositions. Each is grounded in frontline practice yet resists optimisation logics, instead inviting us to think with care as an immanent, situated, and ethically inventive force. These are not reformist tools but speculative invitations born from the event of nursing itself. Having traced how Maria’s minor gestures of care compose a posthuman ethics under pressure, I now turn toward speculation. The propositions below do not aim to fix or improve healthcare systems. Rather, they are conceptual provocations: invitations to imagine how care’s excess might be rendered legible, how systems might accommodate rather than suppress multiplicity. Each is perceptible from the clinical scenario described, not as reformist policy, but as a speculative ethics drawn from the event of nursing itself.

*Proposition 1: Care as Nomadic Speculation:* Clinical documentation systems could include an “assemblage note” field, one hundred characters of free text automatically time‑stamped and visible across disciplines. Instead of forcing Maria to hide her negotiated plan in narrative overflow, the interface itself would invite nomadic annotation: short signals that acknowledge multiplicity (“street‑stable abx dispensed; belongings secured by SG”). These micro-speculations would not replace protocols; they would sit alongside, acting as digital diffraction gratings that allow future clinicians to read patterns of situational difference. Over time, this accumulated diffraction might attune institutions to care for what escapes codification yet sustains practice as excess and remainder.

*Proposition: 2 Adequacy without Totality:* Spinoza’s adequate idea is relational, not exhaustive. Translating that into research design, nursing studies might adopt situated adequacy metrics not as new Key Performance Indicators (KPIs), but as speculative lenses through which to think care’s sufficiency otherwise. For instance, tracking “return-for-follow-up within seven days” could serve as a loose marker of continuity in homeless wound care,not a benchmark to hit, but a way to ask: what counts as staying-with in this ecology? These are not deliverables, but diffractive traces. Potential methods might include: rapid ethnography of after-hours clinics; qualitative audits of shadow documentation; participatory inquiry with patients on what constitutes “enough.” Such approaches embrace incompleteness not as failure, but as an ethical gesture, echoing Barad’s claim that knowing is a “practice of ongoing entanglement” (2007).

*Proposition: 3 Staying with the Trouble for twelve hours at a time:* Imagine a rolling programme of posthuman night rounds: once per fortnight, an inter‑professional trio (nurse, IT analyst, sociologist) shadows the A&E from 19:00 to 07:00, mapping machinic breaks, gathering patient narratives, and tracing conatus vectors across shifts. Fieldnotes are synthesised at dawn into a single‑page “trouble brief.” This brief is not a performance report; it names one machinic break, one assemblage note, and one immanent relation. Over time, these briefs become an archive of nocturnal diffraction that is slow data that does not seek improvement or optimisation but attends to care’s ungovernable textures. This speculative method operationalises Manning’s minor gesture (2016) at the institutional scale, remaking staying with the trouble into an iterative epistemic practice.

Each proposition is modest in scope but radical in implication. They sketch a posthuman nursing praxis that is neither anti‑technology nor techno‑utopian. Instead, they approach nursing as an ongoing experiment in composing with difference. In place of endless binary dropdowns, we cultivate annotated multiplicity; in place of universal KPIs, situated adequacy; in place of distant audits, twelve‑hour co‑inquiry. These are not improvements to the system, but cracks through which other worlds of care might leak.

Had an assemblage note existed, Maria’s decision would still register as deviance in the EPMA logs but this deviance would be legible as generative, an ethically adequate, contextually sufficient act that signalled care beyond the dropdown. A situated adequacy metric would recognise her success when Mr Jones walks back in two days, rather than disappearing. A night‑round team might flag the CDSS IV default as machinic break, not for corrective patching, but to name the interface’s friction with relational ethics. These propositions do not close the argument; they open it. They invite readers (clinicians, designers, theorist) to treat nursing encounters not as sites for performance management but as spaces of speculative events, where posthuman ethics becomes sensible through the practice of care. The event of nursing is not a failure to follow the plan, but an onto‑epistemological force that demands modes of listening, noticing, and naming.

## Nursing as an ongoing event

This paper has proposed that the event of nursing is not a deviation from normative care, but an expression of what care becomes when it exceeds codification, when it moves differently, hesitantly, in response to bodies, relations, histories, and technologies. Beginning with Maria’s minor gesture, a moment of situated deviation from protocol, we followed the ripples of that act through philosophical, technical, and speculative terrains.

Drawing on Spinoza’s relational ontology, Deleuzo-Guattarian assemblage theory, and feminist new materialisms, I argued that the event of nursing is a material-discursive occurrence: an ethics in production in practice, not in abstraction. It is not a rupture, but a recomposition; not an error, but an encounter. Such events are neither rare nor heroic. They happen daily, hourly, in the quiet folds of clinical labour, especially in those moments most likely to be missed by audit systems or algorithmic logics. I reframed deviance not as failure but as generative friction. Through the speculative propositions of Section 4, I gestured toward infrastructural reconfigurations, not to optimise care, but to notice it differently. Assemblage notes, situated adequacy metrics, and twelve-hour inquiries are not recommendations for policy as such; they are experimental devices for staying with the trouble of nursing, for composing with it, for resisting its foreclosure by systems that prefer clarity over complexity.

To take the event of nursing seriously is to ask what it reveals - not about individual nurses, but about the relational, institutional, and machinic forces through which care is performed and governed. It is to hold open space for contingency, inventiveness, and ethical multiplicity. In a time when healthcare is increasingly shaped by datafication and standardisation, I argue that this holding open is itself a vital form of resistance and a call to imagine nursing otherwise.

Rather than conclude, then, I invite. I invite readers to attune and attend to these events: to notice the relational folds, the machinic breaks, the improvisations that do not tidy themselves into protocols but nonetheless sustain lives. The event of nursing is already here; it only asks that we increase our capacities to perceive it.

**References**

Alsaidan, M., Eugene, K., Boyd, A., & Hasan, S. S. (2022). Overrides of electronic medication alerts in hospital practice: A systematic review and meta-analysis. *BMC Medical Informatics and Decision Making, 22*(1), 148.<https://doi.org/10.1186/s12911-022-01881-1>

Barad, K. (2007). *Meeting the universe halfway: Quantum physics and the entanglement of matter and meaning*. Duke University Press.

Bender, M., & Elias, M. (2021). Complexity and care: A relational and systemic account of nursing. *Nursing Philosophy, 22*(1), e12310.<https://doi.org/10.1111/nup.12310>

Bennett, J. (2010). *Vibrant matter: A political ecology of things*. Duke University Press.

Braidotti, R. (2011). *Nomadic subjects: Embodiment and sexual difference in contemporary feminist theory* (2nd ed.). Columbia University Press. (Original work published 1994)

Braidotti, R. (2013). *The posthuman*. Polity Press.

Braidotti, R. (2019). *Posthuman knowledge*. Polity Press.

Browne, A. J., Varcoe, C., Ford-Gilboe, M., & Wathen, C. N. (2015). EQUIP Healthcare: An overview of a multi-component intervention to enhance equity-oriented care in primary health care settings. *International Journal for Equity in Health, 14*, 152.<https://doi.org/10.1186/s12939-015-0271-y>

Cohen, L. (1995). The epistemological politics of cognitive disability. In S. K. Toombs, D. Barnard, & R. A. Carson (Eds.), *Chronic illness: From experience to policy* (pp. 223–237). Indiana University Press.

Deleuze, G. (1988). *Spinoza: Practical philosophy* (R. Hurley, Trans.). City Lights.

Deleuze, G., & Guattari, F. (1987). *A thousand plateaus: Capitalism and schizophrenia* (B. Massumi, Trans.). University of Minnesota Press. (Original work published 1980)

Haraway, D. J. (2016). *Staying with the trouble: Making kin in the Chthulucene*. Duke University Press.

Health Education England. (2019). *The Topol review: Preparing the healthcare workforce to deliver the digital future*.<https://topol.hee.nhs.uk>

Hewett, N., Halligan, A., & Boyce, T. (2012). A general practitioner and nurse led approach to improving hospital care for homeless people. *BMJ, 345*, e5999.<https://doi.org/10.1136/bmj.e5999>

Jackson, A. Y., & Mazzei, L. A. (2012). *Thinking with theory in qualitative research: Viewing data across multiple perspectives*. Routledge.

Lazzarino, R., Parker, C., Wan, J., Higgins, T., & Lewis, D. (2024). Unintended consequences of automated sepsis alerts: A mixed-methods multi-site evaluation in UK emergency departments. *JMIR Human Factors, 11*(2), e53965.<https://doi.org/10.2196/53965>

Lipsky, B. A., Berendt, A. R., Cornia, P. B., Pile, J. C., Peters, E. J. G., Armstrong, D. G., ... & Infectious Diseases Society of America. (2012). 2012 Infectious Diseases Society of America clinical practice guideline for the diagnosis and treatment of diabetic foot infections. *Clinical Infectious Diseases, 54*(12), e132–e173.

Manning, E. (2016). *The minor gesture*. Duke University Press.

Marmot, M., Allen, J., Boyce, T., Goldblatt, P., & Morrison, J. (2020). *Health equity in England: The Marmot Review 10 years on*. Institute of Health Equity.<https://www.instituteofhealthequity.org/resources-reports>

National Institute for Health and Care Excellence. (2015). *Diabetic foot problems: Prevention and management* (NICE guideline NG19).<https://www.nice.org.uk/guidance/ng19>

Nelson, S., & Gordon, S. (2006). *The complexities of care: Nursing reconsidered*. Cornell University Press.

Santos, B. de S. (2014). *Epistemologies of the South: Justice against epistemicide*. Routledge.

Sellman, D. (2011). What makes a good nurse: Why the virtues are important for nurses. *Nursing Philosophy, 12*(1), 28–37.<https://doi.org/10.1111/j.1466-769X.2010.00467.x>

Smith, J., Klumbyte, G., & Britton, R. L. (2023). From ‘if‐then’ to ‘what if?’ Rethinking healthcare algorithmics with posthuman speculative ethics. *Nursing Philosophy, 24*(3), e12447.<https://doi.org/10.1111/nup.12447>

Spinoza, B. (1996). *Ethics* (E. Curley, Trans.). Penguin Books. (Original work published 1677)

Whitehead, A. N. (2014). *The concept of nature*. Cambridge University Press. (Original work published 1920)

Tronto, J. C. (1993). *Moral boundaries: A political argument for an ethic of care*. Routledge.

1. The clinical scenario involving Maria and Mr Jones is a speculative vignette, drawn from patterns commonly observed in emergency care settings. It is not based on a single real case but rather synthesises the recurring dynamics nurses encounter during night shifts. This analytic fiction is used here not to universalise experience but to illuminate the entangled pressures, relations, and ethical demands that constitute the event of nursing (cf. Manning, 2016; Jackson & Mazzei, 2012). [↑](#footnote-ref-1)
